# Missing Value Imputation Approach for Mass Spectrometry-based Metabolomics Data

**DOI:** 10.1038/s41598-017-19120-0

**Published:** 2018-01-12

**Authors:** Runmin Wei, Jingye Wang, Mingming Su, Erik Jia, Shaoqiu Chen, Tianlu Chen, Yan Ni

**Affiliations:** 1 University of Hawaii Cancer Center, Honolulu, HI 96813 USA; 2 Department of Molecular Biosciences and Bioengineering, University of Hawaii at Manoa, Honolulu, HI 96822 USA; 3 Metabo-Profile Biotechnology (Shanghai) Co., Ltd, Shanghai, 201203 P. R. China; 4 Punahou School, Honolulu, HI 96822 USA; 5 Shanghai Key Laboratory of Diabetes Mellitus and Center for Translational Medicine, Shanghai Jiao Tong University Affiliated Sixth People’s Hospital, Shanghai, 200233 China

## Abstract

Missing values exist widely in mass-spectrometry (MS) based metabolomics data. Various methods have been applied for handling missing values, but the selection can significantly affect following data analyses. Typically, there are three types of missing values, missing not at random (MNAR), missing at random (MAR), and missing completely at random (MCAR). Our study comprehensively compared eight imputation methods (zero, half minimum (HM), mean, median, random forest (RF), singular value decomposition (SVD), k-nearest neighbors (kNN), and quantile regression imputation of left-censored data (QRILC)) for different types of missing values using four metabolomics datasets. Normalized root mean squared error (NRMSE) and NRMSE-based sum of ranks (SOR) were applied to evaluate imputation accuracy. Principal component analysis (PCA)/partial least squares (PLS)-Procrustes analysis were used to evaluate the overall sample distribution. Student’s *t*-test followed by correlation analysis was conducted to evaluate the effects on univariate statistics. Our findings demonstrated that RF performed the best for MCAR/MAR and QRILC was the favored one for left-censored MNAR. Finally, we proposed a comprehensive strategy and developed a public-accessible web-tool for the application of missing value imputation in metabolomics (https://metabolomics.cc.hawaii.edu/software/MetImp/).

## Introduction

Metabolomics is the study of systematic identification and/or quantification of wide ranges of small molecule metabolites in bio-samples (cell, tissue, and biological fluids, etc.). Mass spectrometry (MS) is one of the main techniques for metabolomics studies^1^. However, missing values, that certain compounds cannot be identified/quantified in certain samples, occur widely in MS-based metabolomics data. The occurrences of missing values are often caused by biological and/or technical reasons^2,3^. Generally, there are three types of missing values, missing not at random (MNAR), missing at random (MAR), and missing completely at random (MCAR)^4,5^. Unexpected missing values are regarded as MCAR when they originate from random errors and stochastic fluctuations during the data acquisition process (e.g., incomplete derivatization or ionization). MAR assumes the possibility of a variable being missing is determined by other observed variables^4,5^. Thus, missing values due to suboptimal data preprocessing, e.g., inaccurate peak detection and deconvolution of co-eluting compounds, can be called MAR. However, it is hard to distinguish these two types of missing values and some imputation methods can be applied for both MCAR and MAR data^6^. On the other hand, censored missing values caused by under the limits of quantification (LOQ) are considered as MNAR^7^. For example, our previous study showed that bile acids exhibited large variations of concentrations in human serum, targeted identification of a panel of bile acids using MS technique produced many missing values due to LOQ^8^.

The way of handling missing values in metabolomics data differs due to different sources. For those caused by random errors or stochastic fluctuations during data acquisition process, one can re-analyze or re-prepare bio-samples for data acquisition. For missing values produced during the data pre-processing step, filling peaks has been proposed in many tools by simply extracting and replacing with raw or baseline signals, e.g., fillPeaks in XCMS^9^. However, these signals may not be accurate enough to represent real concentration levels of compounds unless baseline has been corrected and none co-eluting compounds exist nearby. For some missing values caused by rigid parameter settings during data processing, one can adjust or apply flexible parameter settings to retrieve real signals of certain compounds, such as signal to noise ratio for peak picking^10^. Missing values due to LOQ (i.e., left censored missing) are usually replaced with a determined small value or zero, which may lead to certain biases, e.g., distortions of the distribution of missing variables and underestimations of the standard deviation^4^. Of course, in metabolomics, missing values that exist in more than 20% of samples may be removed from the data, which is called “80% rule”^2^. To decrease the risk of reducing variable size and losing potential differential metabolites, “modified 80% rule” was proposed that variables can be excluded from the data when the proportion of non-missing elements are accounted for less than 80% among each biological group^11^. On the other hand, instead of removing missing variables directly or replacing missing values with determined values beforehand to prepare a complete dataset for statistical analysis, some new statistical methods have been developed that allow the existence of missing values. Accelerated failure time (AFT) model is based on survival analysis methods, which can be applied directly on left-censored missing datasets^12^. Novel kernel-based methods, i.e., distance-based kernel and stratified kernel, were designed to capture both the continuous pattern and discrete pattern in metabolomics data containing with missing values^13^. However, a complete data is required for most statistical analysis frameworks in metabolomics studies, including PCA and PLS-DA analyses.

Many imputation strategies have been proposed for handling missing values in –omics studies, such as k-nearest neighbors (kNN) imputation^14^, random forest (RF) imputation^15^, and singular value decomposition (SVD) imputation^16^. Several software tools for metabolomics data analysis have implemented different methods dealing with missing values^17–21^. MetaboAnalyst^19,22^, one widely used metabolomics analysis toolkit, provides determined values replacements (e.g., half minimum (HM), mean) and different imputation methods (e.g., Probabilistic PCA (PPCA), Bayesian PCA (BPCA), SVD and kNN). The selection of methods for handling missing values can significantly affect subsequent data analyses and interpretations^23,24^, and it is unclear for users to decide an appropriate one for their data. Gromski *et al*. compared the performance of several missing value imputation methods on GC/MS metabolomics data, including zero, mean, median, kNN, and RF, and recommended RF as a favored one^25^. Guida *et al*. investigated different data processing methods (including normalization, imputation, transformation, and scaling) in non-targeted metabolomics. For imputation, they concluded that RF performed best in PCA and kNN was recommended for partial least squares-discriminant analysis (PLS-DA)^26^. However, these imputation methods they mentioned are suitable for MCAR/MAR only. Hrydziuszko *et al*. raised the importance of selecting optimal methods for treating missing values in metabolomics. They compared eight imputation methods in univariate and multivariate fashions and concluded kNN imputation was an optimal one^3^. Although two types of missing values, MCAR/MAR and MNAR, were mentioned in their work, identical imputation strategies were applied and thus made it unclear to determine suitable methods for different types of missing values. The quantile regression imputation of left-censored data (QRILC), originally proposed for the imputation of MS-based proteomics data, imputes the left-censored missing in truncated fashion could be applied for MNAR in metabolomics^27^. Thus, a comprehensive and systematic evaluation of different methods for handling missing values from different sources is needed for MS-based metabolomics studies.

In this study, we considered both MCAR/MAR and left-censored MNAR data in two separate clinical metabolomics studies, and compared five different imputation methods (i.e., RF, kNN, SVD, Mean, Median) for MCAR/MAR and six imputation methods (i.e., QRILC, Half-minimum, Zero, RF, kNN, SVD) for MNAR. Then, we systematically measured the performance of those imputation methods using three different ways: (1) normalized root mean squared error (NRMSE) and NRMSE-based sum of ranks (SOR) were applied to evaluate the imputation accuracy for MCAR/MAR and MNAR correspondingly; (2) principal component analysis (PCA)/partial least squares (PLS)-Procrustes sum of squared error were used to evaluate the overall sample distribution; and (3) student’s *t*-test followed by Pearson correlation analysis was conducted to evaluate the effect of imputation on univariate statistical analysis. Results showed that RF imputation performed the best for MCAR/MAR and QRILC was the favored one for MNAR. However, with the increasing number of missing values, the imputation performances will decrease drastically. Additionally, we evaluated the types of missing values in two different real metabolomics datasets, and found MCAR/MAR widely occurred in GC/MS profiling data and MNAR existed in LC/MS targeted data. Finally, taking account of removing missing variables that contain big proportions of missing values beforehand, we proposed a comprehensive strategy and a public-accessible web-tool for the public to deal with missing values in metabolomics studies (https://metabolomics.cc.hawaii.edu/software/MetImp/).

## Results

### MCAR/MAR imputation and evaluation

**Figure 1:**
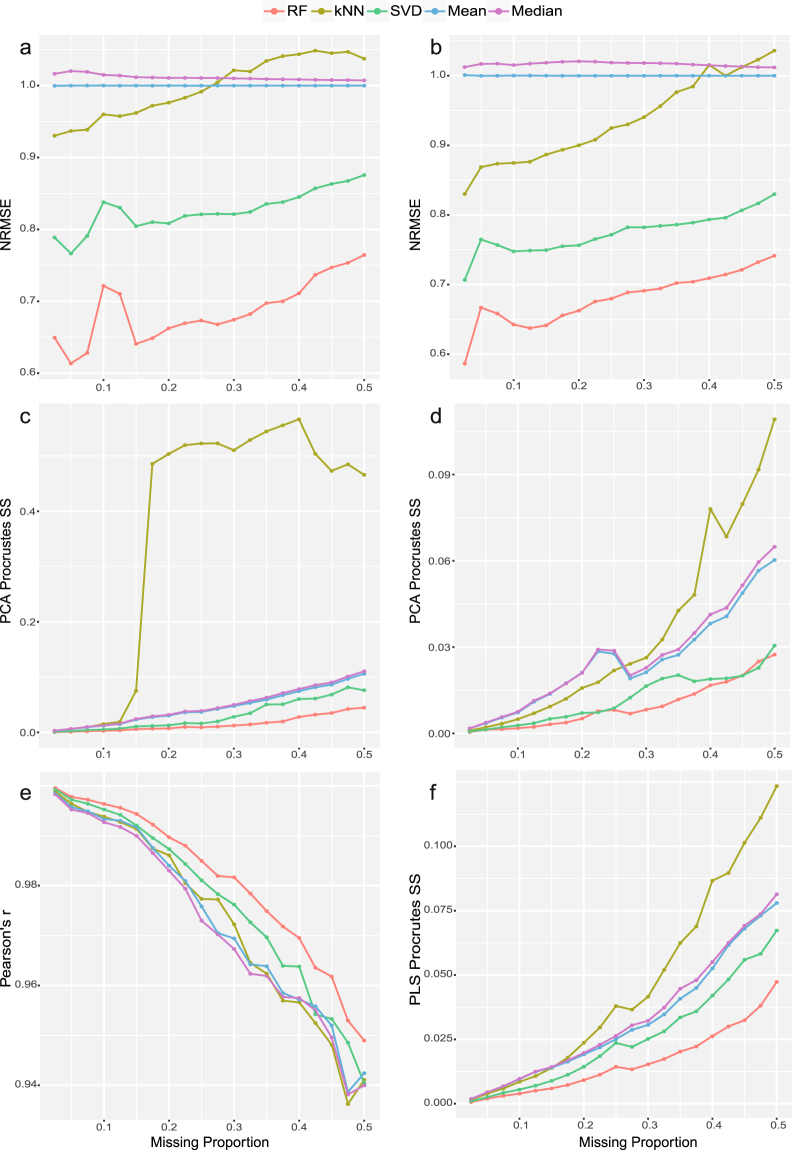
Evaluation of different imputation methods for MCAR/MAR (**a,b**) NRMSE on unlabeled and labeled metabolomics data. (**c,d**) PCA-Procrustes sum of squared errors on unlabeled and labeled metabolomics data. (**e**) Pearson correlation of log *p*-values (*t*-test) of complete data and imputed data. (**f**) PLS-Procrustes sum of squared errors.

We generated random missing values on first two data sets from the proportion of 2.5% to 50% in step of 2.5%. Five different imputation methods, RF, kNN, SVD, Mean and Median were conducted on all missing data sets. After imputations, NRSME were calculated by comparing differences between imputed data and complete data after z-score transformation (scaling and centralization). Z-score transformation makes it an unbiased comparison of using NRMSE considering the different ranges of variables in their original levels where the variable with large values will dominate the evaluation. Fig. 1a,b showed that RF imputation performed the best on both data sets with different proportions of missing values, followed by SVD and kNN imputation. We also found that kNN imputation method produced even larger NRMSE than two determined value imputation methods (i.e., mean and median) when the missing proportion increased to certain points. In contrast, these two determined value imputations performed stably on data with different proportions of missing values since the imputed “average” values made the mean squared error, the numerator of formula (1), equals/close to the denominator which is the variance of missing elements in complete data.

Next, we applied PCA to both complete and imputed data sets and selected first two PCs as they represented the most variance. Then, we applied Procrustes analysis to compare the distribution of sample points on top two PCs of imputed data with the complete data using scaled sum of squared errors. Less distortion of imputed data (smaller sum of squared errors) represented a better recovery of imputation regarding the original sample distribution. Results showed that RF performed the best across different missing proportions on both data sets (Fig. 1c,d). Meanwhile, these two determined value imputation methods, mean and median, gave very similar trends. In contrast, kNN started performing the worst once the proportion of missing values reached to certain cutoff values.

With sample phenotype information (case/control), we performed both Student’s *t*-test and PLS-DA on the second data set with original values or imputed values. First, we conducted student’s *t*-test on each variable. Then, we measured the Pearson’s *r* between the log-transformed *p*-values calculated from imputed data and complete data. Results showed that RF imputation maintained the highest correlation coefficients across different missing proportions (Fig. 1e), which indicated that the most information of original univariate results had been remained using RF method. In addition, we applied PLS-Procrustes analysis and found that RF imputation kept the best among five imputation methods with the lowest sum of squared errors (Fig. 1f).

### MNAR imputation and evaluation

For each MNAR data set, six different imputation methods were applied with two aims: first, to evaluate the performances of those imputation methods previously applied on MCAR/MAR (i.e., RF, kNN, SVD) on the situation of MNAR; second, to compare the performance of three left-censored imputation methods (i.e., QRILC, HM and Zero) on MNAR. After imputation, we introduced NMRSE-based SOR as a non-parametric method to evaluate their imputation accuracy considering the skewed distribution of MNAR dataset. The rationale of applying SOR instead of NMRSE has been explained in supporting information (Fig. S7–S8).

**Figure 2:**
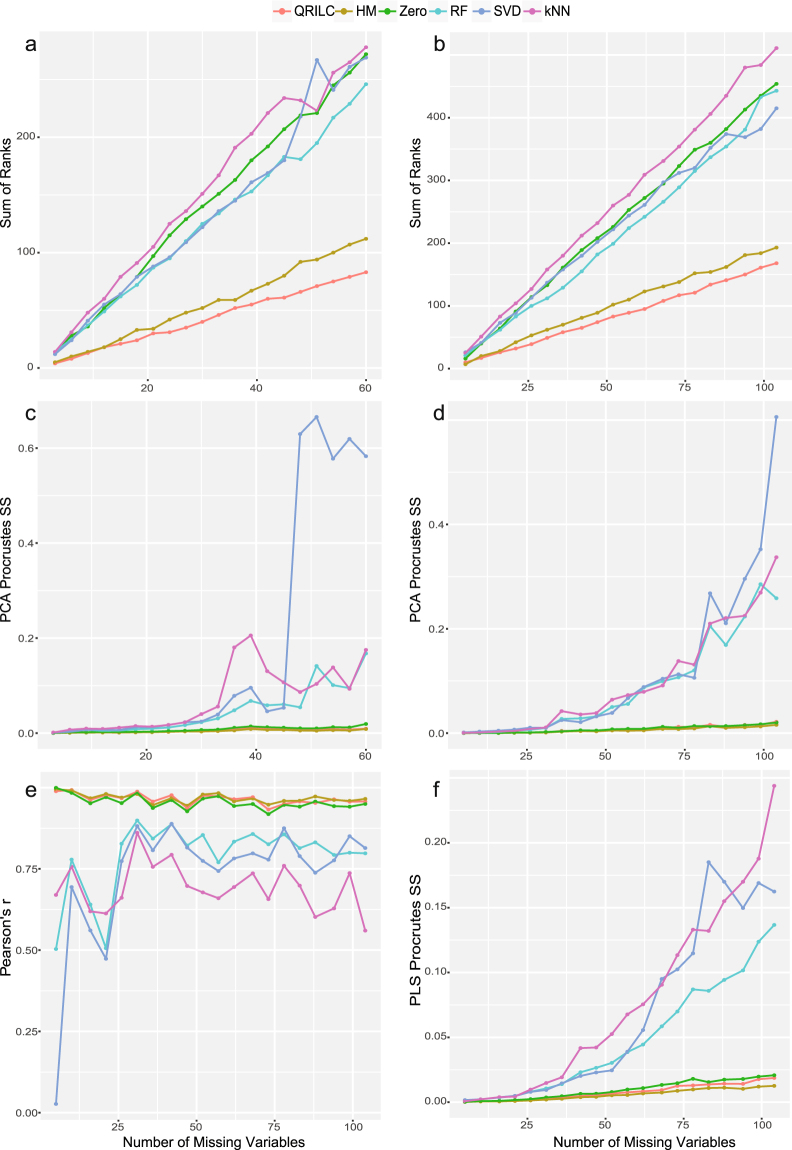
Evaluation of different imputation methods for MNAR (**a,b**) SOR on unlabeled and labeled metabolomics data. (**c,d**) PCA-Procrustes sum of squared errors on unlabeled and labeled metabolomics data. (**e**) Pearson correlation of log *p*-values (*t*-test) of missing variables from complete data and imputed data. (**f**) PLS-Procrustes sum of squared errors.

Results of SOR (Fig. 2a,b) showed that all three imputation methods, RF, SVD, and kNN, performed poorly on MNAR, together with Zero imputation that had been commonly used in metabolomics data analysis. In comparison, QRILC produced much smaller SOR values followed by HM imputation, showing consistent good performances on data with different numbers of missing variables.

From the PCA-Procrustes analysis, we observed that three imputation methods for MCAR/MAR changed original sample distribution to a large extent as the number of missing variables increased (Fig. 2c,d). In contrast, three MNAR imputation methods showed consistent results, among which, HM performed slightly better across the different number of missing variables followed by QRILC.

For the correlation analysis on log *p*-values of missing variables, Fig. 2e also demonstrated that three MNAR imputation methods performed better with higher correlation coefficients and QRILC performed almost as good as HM. For the PLS-Procrustes analysis (Fig. 2f), the result was similar to PCA-Procrustes analysis that we observed from Fig. 2d. To summarize, both QRILC and HM showed decent and stable performances on MNAR with different numbers of missing variables.

### QRILC and HM imputation

**Figure 3:**
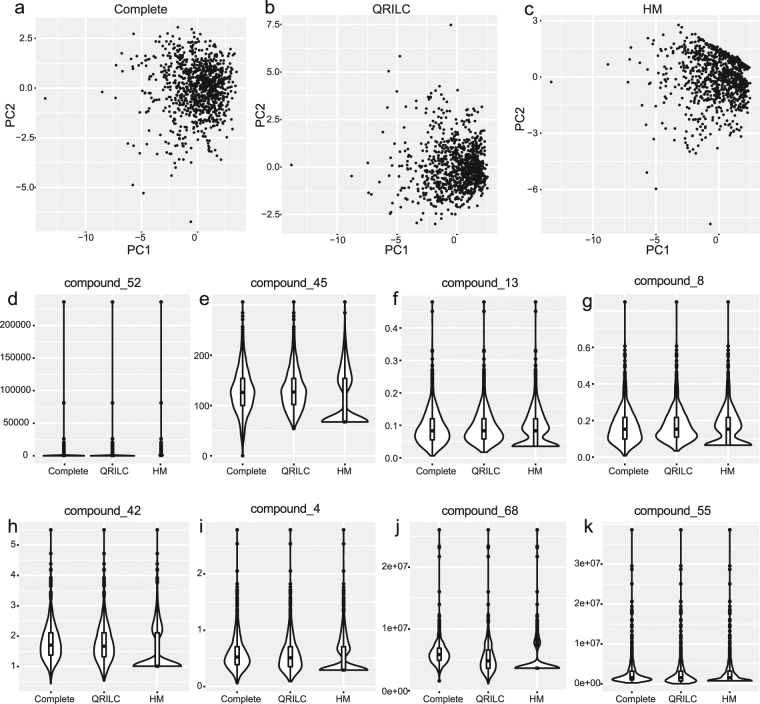
Comparisons between QRILC and HM for MNAR (**a-c**) PCA score plot for complete data, QRILC imputed data and HM imputed data on top 2 PCs. (**d–k**) Violin plots of eight missing variables.

Next, we further compared the overall performance of HM and QRILC methods. QRILC imputes the left-censored data by randomly drawing values from a truncated normal distribution while HM replaces missing elements by using the half of the minimum of non-missing values. As a determined value imputation method, HM has limitations in some circumstances (e.g., distorting distributions and underestimating the variances of missing variables that further affect multivariate analysis)^4^. In this work, we randomly selected ten variables from the first data set to construct a new data set and assigned eight of them as missing variables which contained 40~80% left-censored missing values. PCA analysis on complete data, QRILC imputed, and HM imputed data with top 2 PCs showed that QRILC kept the overall shape and distribution of original data set while HM showed a subgroup of samples gathering around to a straight line (Fig. 3a–c). This was because missing values of those samples were replaced by the same determined values, making those samples gather towards a straight line on the score plot after linear transformation using PCA. In addition, the violin plots also demonstrated that HM method severely distorted the distributions of eight variables (Fig. 3d–k).

### Missing types in metabolomics data

To investigate different missing types in metabolomics data, we further evaluated two extra datasets. For the GC/MS untargeted data, there existed 317 missing values among original dataset. We have examined each missing value and can manually re-identify 221 of them. These missing values occurred during different steps of data preprocessing, including peak picking of compounds with low signal to noise ratios, deconvolution of co-eluting compounds with high mass spectral similarities, alignment for certain compounds with large retention time shifts. As a result, those retrieved values of missing elements are randomly distributed among original dataset and not related to their true abundances in general (Fig. S12a). After comparing different imputation methods on this data, RF outperformed other four methods using both NRMSE and PCA-Procrustes analysis (Table S20). In the LC/MS targeted data set, we can manually re-identified 26 missing values and obtained their original responses from raw data, while the rest of them were missing due to the limit of detection. When we compared the responses of re-identified 26 missing elements with non-missing elements, missing values were abundance-dependent since response values of missing part are significantly lower than the non-missing part (Fig. S12b). In addition, QRILC and HM exceeds other methods on SOR and QRILC has the best performance on PCA-Procrustes analysis (Table S21). Based on these findings, we summarized that MCAR/MAR widely occurred in non-targeted GC/MS metabolomics data and left-censored MNAR existed in targeted LC/MS metabolomics dataset.

## Discussion

To avoid potentially biased comparisons, we have explored the performance of each imputation method for MS-based metabolomics data and optimized parameter settings or data pre-processing steps to reach optimal performance. For example, kNN was previously applied on a *p* × *n* gene expression matrix (genes in rows and samples in columns) while we found kNN performed better on an *n* × *p* metabolomics data matrix (samples in rows and metabolites in columns). The underlying difference is that instead of finding k nearest variables, which are genes in their original case, we tried to find k nearest samples to represent the missing ones. This is reasonable since gene expression data usually contains a large number (more than 10,000) of genes where co-expression occurs frequently thus neighbored genes are usually good representation for missing ones. For SVD imputation, as suggested by the original paper, a pre-scaling of the data will increase the accuracy, and we also found using top five PCs is a good choice in our study rather than the default setting of two. Since the original scale function in R is irreversible, we implement a scale-recover function, which enables us to recover the scaled table to the original scale with parameters recorded in the scaling process. For QRILC imputation, log-transformation beforehand was found useful not only to improve imputation accuracy but also to ensure a positive value in the original scale.

In this work, we systematically evaluated a total of eight imputation methods on different types of missing values in terms of imputation accuracy, sample distribution, and statistical analysis. Results showed that RF imputation performed the best for MCAR/MAR data. For left-censored MNAR, we found that QRILC and HM performed better than others, however, HM could distort the distribution of single variables as indicated by violin plots or of a linear combination of variables as indicated by PCA score plots. Thus, we recommend the more smoothed method QRILC for left-censored MNAR. Additionally, we investigated the types of missing values usually occur in GC/MS profiling and targeted LC/MS metabolomics studies. We found MCAR/MAR widely occurred in GC/MS profiling data due to data preprocessing and left-censored MNAR in targeted LC/MS data due to the limit of detection and/or quantitation.

**Figure 4:**
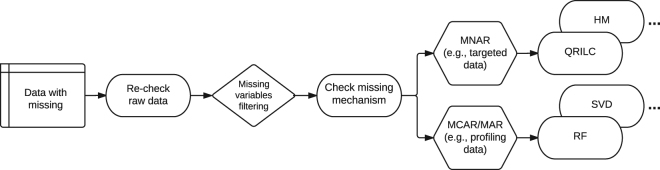
An imputation strategy for metabolomics studies.

In general, with the increasing number of missing values, the imputation performances will decrease severely. Thus, taking account of removing missing variables beforehand that contain big proportions of missing values, we proposed a comprehensive strategy to deal with missing values in metabolomics studies (Fig. 4). For both targeted and profiling/non-targeted metabolomics data, we recommend (1) checking raw data and if necessary, adjusting parameter settings or manually peak matching to fill back certain missing values in an accurate way; (2) filtering missing variables, e.g. “modified 80% rule”, to remove those unreliable variables with big proportion of missing values; (3) for those missing values that cannot be retrieved, users need to assess the possible reasons of producing missing values and choose an appropriate one for missing value imputation; (4) for left-censored MNAR situations (e.g., in targeted metabolomics data), QRILC imputation is recommended; and RF imputation is recommended for MCAR/MAR situations (e.g., in profiling/non-targeted metabolomics data).

In addition, a web-tool (https://metabolomics.cc.hawaii.edu/software/MetImp/) has been developed allowing users to upload their data and choose an appropriate method for missing value imputation. As described in this work, eight different methods dealing with missing values are provided in the web server. “Modified 80% rule” (0%~100% adjustable) was the default setting for group-wise/non-group-wise missing filtering while RF and QRILC were provided as default imputation methods corresponding to MCAR/MAR and MNAR. Additionally, all settings are interactively modifiable for the flexibility of usage. Finally, a completed dataset with imputed missing values is generated and can be downloaded for further statistical analyses.

Since the process of evaluating different missing value imputation methods is also important, we have packaged our evaluation processes into single R functions, which integrates batch generation of missing values (a range of missing proportions), imputation (different pre-defined and customized imputation functions), evaluation (NRMSE, SOR, correlation, PCA/PLS-Procrustes analysis), and visualization with flexible parameter settings. The comprehensive imputation evaluation pipeline can be accessible from GitHub (https://github.com/WandeRum/MVI-evaluation). This evaluation pipeline enables researchers to perform further studies on missing value imputation problems, e.g., comparing new imputation methods with existing ones, and evaluating different methods on specific data sets. In the future, new methods dealing with metabolomics missing values, especially for MNAR, will be introduced for comprehensive comparison through our evaluation pipeline and added to our web-tool. It is also valuable to investigate the types of missing values present in different metabolomics studies, especially targeted GC/MS dataset and untargeted LC/MS dataset.

## Methods

### Metabolomics data sets

A total number of four real-world metabolomics data sets were included in this study. The first two were applied to evaluate the performance of different imputation methods. Since the measurements required comparisons between imputed data and original data, a complete raw data set was needed in our studies. Thus, we removed all missing values in our original data beforehand and left a complete data set for consequential analysis.

1. This data set includes a total of 977 de-identified subjects and 75 metabolites without missing values. These metabolites include free fatty acids, amino acids, and bile acids, which were identified using both GC/MS-based non-targeted analysis and LC/MS-based targeted metabolomics approach. It served as a large sample size data set for label-free evaluation.
2. This data set was collected from a study of comparing metabolic profiles between obese subjects with diabetes mellitus and healthy controls^28,29^. After filtering all missing values, this data set contained a total number of 198 subjects (70 patients, 128 healthy controls) and 130 metabolites. These metabolites include free fatty acids, amino acids, and bile acids that were identified using LC/MS-based targeted metabolomics approaches. It served as medium sample size data set for both label-free and labeled data evaluation.
  Then the other two datasets with missing elements were applied to determine the types of missing values present in different metabolomics datasets.
3. The is a GC/MS profiling data that contains 37 samples and 110 metabolites identified, with 317 missing values and 221 of them were re-identified manually.
4. This is a targeted LC/MS metabolomics dataset, which includes 40 samples and 41 metabolites, with 88 missing elements and 26 of them were re-identified manually.

### Missing value generation

For MCAR/MAR generation, we randomly drew elements and replaced with missing values (NA) from the complete data matrix across the proportions from 2.5% to 50% in a step of 2.5% to generate a list of missing data sets.

For MNAR generation, the process could be divided into two steps. First, we randomly selected a proportion/number (from 4% to 80% in a step of 4%) of metabolites as missing variables that will contain missing values. For example, 4% means we randomly picked 4 variables out of 100 as missing variables. Since missing values usually occur in a group of metabolites with low concentration/response detected from analytical instruments, the first step allows missing values occurring only in a group of variables in a similar way. Then, we generated a random quantile cut off from the range 30%~60% uniformly for each missing variable and replaced those elements under the cutoff with missing values. For example, if cutoff = 0.5, then all values lower than the median point in this variable will be replaced with NA values.

### Missing value imputation methods

For the situation of MCAR/MAR, we applied five different imputation methods, including kNN, RF, SVD, Mean, and Median. For the situation of MNAR, we applied kNN, RF, SVD, QRILC, Zero, and HM. We performed all the data analysis using R language (Version 3.4.2)^30^.

- **kNN** (k Nearest Neighbors Imputation)^14^: The original kNN imputation was developed for high-dimensional microarray gene expression data (n «p, n is the number of samples, and p is the number of variables). For each gene with missing values, this method finds the k nearest genes using Euclidean metric and imputes missing elements by averaging those non-missing elements of its neighbors. In metabolomics studies, we applied kNN to find k nearest samples instead and imputed the missing elements. We applied R package *impute* for this imputation approach.
- **RF** (Imputation with Random Forest)^15^: This imputation method applies random forest, a powerful machine learning algorithm, to build a prediction model by setting particular target variable with non-missing values as the outcome and other variables as predictors, then to predict the target variable with missing values iteratively. The R package *missForest* was used for this approach.
- **SVD** (Singular Value Decomposition Imputation)^16,31^: SVD imputation will initialize all missing elements with zero then estimate them as a linear combination of the k most significant eigen-variables iteratively until reaches certain convergence threshold. In metabolomics data, we scaled and centralized the data matrix first and then applied this imputation approach with the number of PCs setting to five by using R package *pcaMethods*.
- **Mean**: This method replaces missing elements with an average value of non-missing elements in the corresponding variable.
- **Median**: This method replaces missing elements with a median value of non-missing elements in the corresponding variable.

- **QRILC** (Quantile Regression Imputation of Left-Censored data)^27^: QRILC imputation was specifically designed for left-censored data, data missing caused by lower than LOQ. This method imputes missing elements with randomly drawing from a truncated distribution estimated by a quantile regression. A beforehand log-transformation was conducted to improve the imputation accuracy. R package *imputeLCMD* was applied for this imputation approach.
- **Zero**: This method replaces all missing elements with zero.
- **HM** (Half of the Minimum): This method replaces missing elements with half of the minimum of non-missing elements in the corresponding variable.

### Performance evaluation

Normalized Root Mean Squared Error (NRMSE) has been commonly used to evaluate accuracy by calculating the differences between imputed values and real values by following formula^32^

1 $$NRMSE=\sqrt{\frac{mean({({X}^{true}-{X}^{imp})}^{2})}{var({X}^{true})}}$$

where *X*^*true*^ is the true data, *X*^*imp*^ is the imputed data. We calculated NRMSE for the situation of MCAR/MAR on scaled data.

For MNAR, considering the missing value is not randomly distributed, using NRMSE directly might cause biased results especially for those imputation methods with determined values as we showed in the Supplements. Thus, we derived another metric, which was NRMSE-based sum of ranks (SOR). We first calculated the NRMSE for each missing variable and ranked them across different imputation methods. Consequently, we summed the rank of all missing variables for each method and made a comparison based on SOR. The SOR can be represented as following formula

2 $$SOR=\sum _{i=1}^{M}Ran{k}_{i}(NRMSE)$$

where M is the number of missing variables, *Rank*_*i*_(*NRMSE*) means the rank of NRMSE of different imputation methods in *i*_*th*_ missing variable. This non-parametric measurement provides a robust and unbiased comparison especially for the situation of MNAR.

NRMSE and SOR measured imputation accuracy in respect of the value of missing elements. Additionally, we measured the effects of different imputation methods in respect of overall sample distribution. To do this, we first applied the dimension reduction approach, e.g. PCA, to reduce the data dimensions. Then, Procrustes analysis, a statistical shape analysis, was conducted to compare the alteration of the imputed sample distributions with original sample distribution in the space of top PCs. Procrustes sum of squared errors were calculated as a quantitative measurements. R package *vegan* was applied for Procrustes analysis^33^.

In addition to the above label-free evaluations, we conducted extra evaluations from a statistical analysis perspective on the labeled data. These evaluations are mainly focused on the influences of different imputation methods on consequential statistical analyses. For univariate analysis, we conducted Student’s *t*-test on variables from imputed data and original data and *p*-values were then log-transformed considering their skewed distribution. We then conducted Pearson correlation analysis on the log *p*-values of imputed data and original data. For multivariate analysis, one of the most widely used methods in metabolomics studies is partial least squared regression (PLSR)/discriminant analysis (PLS-DA). In our case, since the phenotype outcome variable was case/control, we applied PLS-DA as a supervised dimensional reduction approach first. Then, we conducted Procrustes analysis to compare the sample distribution between imputed data and original data as we did previously. R package *ropls* was applied for PLS-DA^34^.

### Data availability

The data set (2) from a diabetes study is available on both of our web-server (https://metabolomics.cc.hawaii.edu/software/MetImp/) and GitHub (https://github.com/WandeRum/MVI-evaluation). Other data sets are available upon request.

### Associated Content

Supplements.docx – Imputation Evaluation and Visualization Vignette.

## Electronic supplementary material

Supplementary Information
