# Supplementary material for: Missing Value Imputation Approach for Mass Spectrometry-based Metabolomics Data
Source: Sci Rep. 2018 Jan 12;8:663. doi: 10.1038/s41598-017-19120-0 (PMC5766532; doi:10.1038/s41598-017-19120-0)
Supplement: Supplementary file 1 — Supplementary Information [file 41598_2017_19120_MOESM1_ESM.doc]

# Supplementary information:

**Missing Value Imputation Approach for Mass Spectrometry-based Metabolomics Data**

Runmin Wei^1,2,#^, Jingye Wang^1,#^, Mingming Su^1,3^, Erik Jia^4^, Shaoqiu Chen^1,2^, Tianlu Chen^5^, Yan Ni^1,*^

^1^ University of Hawaii Cancer Center, Honolulu, HI 96813, USA

^2^ Department of Molecular Biosciences and Bioengineering, University of Hawaii at Manoa, Honolulu, HI 96822, USA

^3^ Metabo-Profile Biotechnology (Shanghai) Co., Ltd., Shanghai 201203, P.R.China

^4^ Punahou School, Honolulu, HI 96822, USA

^5^ Shanghai Key Laboratory of Diabetes Mellitus and Center for Translational Medicine, Shanghai Jiao Tong University Affiliated Sixth People’s Hospital, Shanghai 200233, China

^#^ These authors contributed equally to this work.

^*^ Corresponding Author: Yan Ni, PhD, Telephone: 808-564-5886, Fax: 808-586-2970, Email: yni@cc.hawaii.edu

#

# Imputation evaluation and visualization vignette

## Package install and data import

library(magrittr)
library(knitr)
library(ggplot2)
library(reshape2)
source('Imputation evaluations.R')
data_test <- read.csv('OB_data/Real_data_DM.csv', row.names = 1)
group <- rownames(data_test) %>% gsub('()-.*', '\\1', .) %>% as.factor()

**Table S1: An overview of example dataset**

|  | var_1 | var_2 | var_3 | var_4 | var_5 | var_6 | var_7 | var_8 |
| --- | --- | --- | --- | --- | --- | --- | --- | --- |
| DM-1 | 70.791 | 514.177 | 206.219 | 411.484 | 4.721 | 21.626 | 7.300 | 0.648 |
| DM-10 | 50.623 | 128.610 | 41.453 | 227.880 | 0.540 | 47.970 | 4.781 | 2.123 |
| DM-11 | 42.720 | 125.704 | 46.343 | 160.441 | 2.990 | 9.155 | 9.994 | 0.524 |
| DM-12 | 15.682 | 67.553 | 21.916 | 71.591 | 0.728 | 23.039 | 5.737 | 2.506 |
| DM-13 | 65.920 | 72.615 | 18.242 | 123.881 | 1.381 | 18.932 | 3.326 | 0.708 |
| DM-14 | 161.750 | 379.706 | 26.088 | 274.699 | 0.490 | 43.720 | 15.053 | 4.504 |

## [1] DM DM DM DM DM DM DM DM DM DM DM DM DM DM DM DM DM DM DM DM DM DM DM
## [24] DM DM DM DM DM DM DM DM DM DM DM DM DM DM DM DM DM DM DM DM DM DM DM
## [47] DM DM DM DM DM DM DM DM DM DM DM DM DM DM DM DM DM DM DM DM DM DM DM
## [70] DM N N N N N N N N N N N N N N N N N N N N N N
## [93] N N N N N N N N N N N N N N N N N N N N N N N
## [116] N N N N N N N N N N N N N N N N N N N N N N N
## [139] N N N N N N N N N N N N N N N N N N N N N N N
## [162] N N N N N N N N N N N N N N N N N N N N N N N
## [185] N N N N N N N N N N N N N N
## Levels: DM N

# MCAR

## MCAR generation and imputation

MCAR_list <- MCAR_gen_imp(data_c = data_test, prop = seq(.1, .5, .1), impute_list = c('kNN_wrapper', 'SVD_wrapper', 'Mean_wrapper', 'Median_wrapper', 'RF_wrapper'), cores = 10)

## MCAR NRMSE evaluation and plot

MCAR_NRMSE_list <- NRMSE_cal_plot(MCAR_list, plot = T, x = 'Miss_Prop')

## [1] 1
## [1] 2
## [1] 3
## [1] 4
## [1] 5


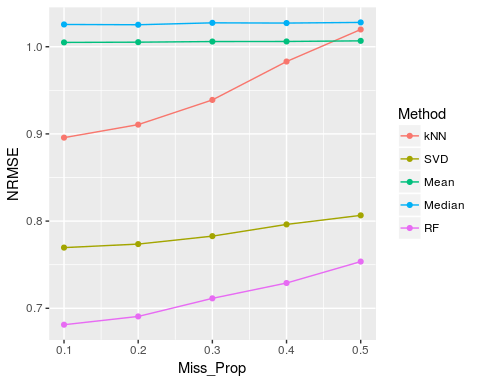


**Figure S1:** NRMSE for 5 different imputation methods on MCAR. X-axis indicates the proportion of missing values and y-axis is the calculated NRMSE.

**Table S2:** The NRMSE of 5 different imputation methods on MCAR

| kNN | SVD | Mean | Median | RF | Miss_Prop | Miss_Num |
| --- | --- | --- | --- | --- | --- | --- |
| 0.8957382 | 0.7696032 | 1.004958 | 1.025544 | 0.6811728 | 0.1 | 130 |
| 0.9106655 | 0.7736548 | 1.005224 | 1.025213 | 0.6906557 | 0.2 | 130 |
| 0.9389917 | 0.7827661 | 1.005945 | 1.027410 | 0.7113980 | 0.3 | 130 |
| 0.9830233 | 0.7961935 | 1.006039 | 1.027144 | 0.7289833 | 0.4 | 130 |
| 1.0197809 | 0.8065801 | 1.006747 | 1.027885 | 0.7535703 | 0.5 | 130 |

**Table S3:** The melted table of Table S2

| Miss_Prop | Method | NRMSE |
| --- | --- | --- |
| 0.1 | kNN | 0.8957382 |
| 0.2 | kNN | 0.9106655 |
| 0.3 | kNN | 0.9389917 |
| 0.4 | kNN | 0.9830233 |
| 0.5 | kNN | 1.0197809 |
| 0.1 | SVD | 0.7696032 |

The above melted table is for ggplot2

## PCA Procrustes analysis on MCAR

MCAR_PCA_ProSS_list <- Procrustes_cal_plot(MCAR_list, DR = 'PCA', nPCs = 2, x = 'Miss_Prop', plot = T)

## [1] 1
## [1] 2
## [1] 3
## [1] 4
## [1] 5


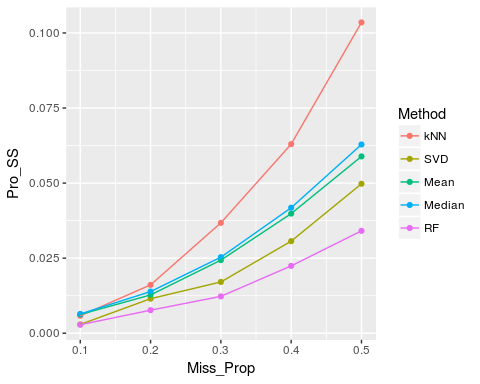


**Figure S2:** The PCA-Procrustes Sum of Squared Error of 5 different imputation methods on MCAR. X-axis indicates the proportion of missing values and y-axis is the PCA-Procrustes Sum of Squared Error.

**Table S4:** The PCA-Procrustes Sum of Squared Error of 5 different imputation methods on MCAR

| kNN | SVD | Mean | Median | RF | Miss_Prop | Miss_Num |
| --- | --- | --- | --- | --- | --- | --- |
| 0.0058081 | 0.0029227 | 0.0062499 | 0.0064181 | 0.0028319 | 0.1 | 130 |
| 0.0160971 | 0.0115052 | 0.0127537 | 0.0138729 | 0.0076673 | 0.2 | 130 |
| 0.0367555 | 0.0170723 | 0.0243381 | 0.0253273 | 0.0122743 | 0.3 | 130 |
| 0.0629755 | 0.0306623 | 0.0398333 | 0.0417957 | 0.0224246 | 0.4 | 130 |
| 0.1035155 | 0.0497668 | 0.0588806 | 0.0628540 | 0.0340962 | 0.5 | 130 |

**Table S5:** The melted table of Table S4

| Miss_Prop | Method | Pro_SS |
| --- | --- | --- |
| 0.1 | kNN | 0.0058081 |
| 0.2 | kNN | 0.0160971 |
| 0.3 | kNN | 0.0367555 |
| 0.4 | kNN | 0.0629755 |
| 0.5 | kNN | 0.1035155 |
| 0.1 | SVD | 0.0029227 |

The above melted table is for ggplot2

## MCAR T-test results correlation

MCAR_Ttest_cor_list <- Ttest_cor_cal_plot(MCAR_list, group = group, plot = T, x = 'Miss_Prop', cor = 'P')

## [1] 1
## [1] 2
## [1] 3
## [1] 4
## [1] 5


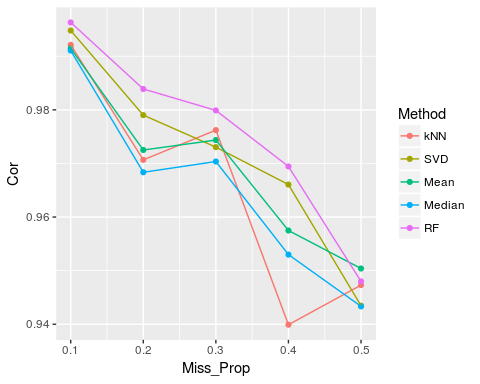


**Figure S3:** The Pearson Correlation of log-transformed P-values from student t-test between imputed data and complete data on MCAR. X-axis is the missing proportion and y-axis is the Pearson correlation.

**Table S6:** The Pearson Correlation of log T-test P-values between imputed data and complete data on MCAR.

| kNN | SVD | Mean | Median | RF | Miss_Prop | Miss_Num |
| --- | --- | --- | --- | --- | --- | --- |
| 0.9921166 | 0.9947967 | 0.9914626 | 0.9910585 | 0.9963095 | 0.1 | 130 |
| 0.9706710 | 0.9790125 | 0.9725072 | 0.9683502 | 0.9838864 | 0.2 | 130 |
| 0.9762177 | 0.9730374 | 0.9743628 | 0.9703540 | 0.9798917 | 0.3 | 130 |
| 0.9399089 | 0.9660529 | 0.9574841 | 0.9529879 | 0.9694339 | 0.4 | 130 |
| 0.9473047 | 0.9434554 | 0.9503873 | 0.9433293 | 0.9479859 | 0.5 | 130 |

**Table S7:** The Spearman Correlation of T-test P-values between imputed data and complete data on MCAR.

| kNN | SVD | Mean | Median | RF | Miss_Prop | Miss_Num |
| --- | --- | --- | --- | --- | --- | --- |
| 0.9906485 | 0.9914788 | 0.9890754 | 0.9883762 | 0.9939915 | 0.1 | 130 |
| 0.9803247 | 0.9781016 | 0.9776427 | 0.9761952 | 0.9877699 | 0.2 | 130 |
| 0.9700610 | 0.9571263 | 0.9714758 | 0.9686299 | 0.9732510 | 0.3 | 130 |
| 0.9372598 | 0.9338513 | 0.9505933 | 0.9454861 | 0.9560120 | 0.4 | 130 |
| 0.9098718 | 0.9009081 | 0.9193762 | 0.9063868 | 0.9426621 | 0.5 | 130 |

## PLS Procrustes analysis on MCAR

MCAR_PLS_ProSS_list <- Procrustes_cal_plot(MCAR_list, DR = 'PLS', nPCs = 2, outcome = group, x = 'Miss_Prop', plot = T)

## [1] 1
## [1] 2
## [1] 3
## [1] 4
## [1] 5


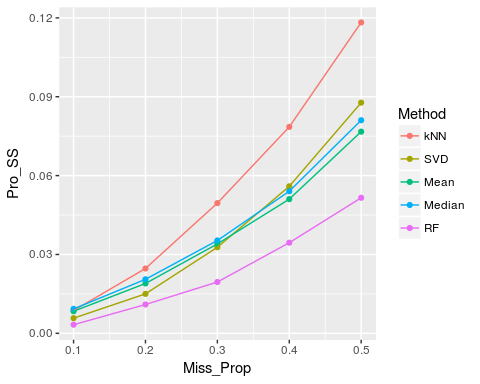


**Figure S4:** The PLS-DA-Procrustes Sum of Squared Error of 5 different imputation methods on MCAR. X-axis is the missing proportion and y-axis is the PLS-Procrustes Sum of Squared Error.

**Table S8:** The PLS-DA-Procrustes Sum of Squared Error of 5 different imputation methods on MCAR

| kNN | SVD | Mean | Median | RF | Miss_Prop | Miss_Num |
| --- | --- | --- | --- | --- | --- | --- |
| 0.0086773 | 0.0057721 | 0.0084034 | 0.0092919 | 0.0032826 | 0.1 | 130 |
| 0.0246732 | 0.0150190 | 0.0189715 | 0.0205573 | 0.0109688 | 0.2 | 130 |
| 0.0495326 | 0.0327509 | 0.0340160 | 0.0353257 | 0.0195211 | 0.3 | 130 |
| 0.0785230 | 0.0559197 | 0.0510504 | 0.0540521 | 0.0344740 | 0.4 | 130 |
| 0.1182722 | 0.0877537 | 0.0767151 | 0.0810655 | 0.0515663 | 0.5 | 130 |

**Table S9:** The melted table of Table S8

| Miss_Prop | Method | Pro_SS |
| --- | --- | --- |
| 0.1 | kNN | 0.0086773 |
| 0.2 | kNN | 0.0246732 |
| 0.3 | kNN | 0.0495326 |
| 0.4 | kNN | 0.0785230 |
| 0.5 | kNN | 0.1182722 |
| 0.1 | SVD | 0.0057721 |

# MNAR

## MNAR generation and imputation

MNAR_list <- MNAR_gen_imp(data_c = data_test, mis_var_prop = seq(.1, .7, .1), var_mis_prop = seq(.3, .6, .1), impute_list = c('kNN_wrapper', 'SVD_wrapper', 'QRILC_wrapper', 'HM_wrapper', 'Zero_wrapper'), cores = 1)

## MNAR NRMSE evaluation and plot

MNAR_NRMSE_list <- NRMSE_cal_plot(MNAR_list, plot = T, x = 'Miss_Num', sc=F)

## [1] 1
## [1] 2
## [1] 3
## [1] 4
## [1] 5
## [1] 6
## [1] 7


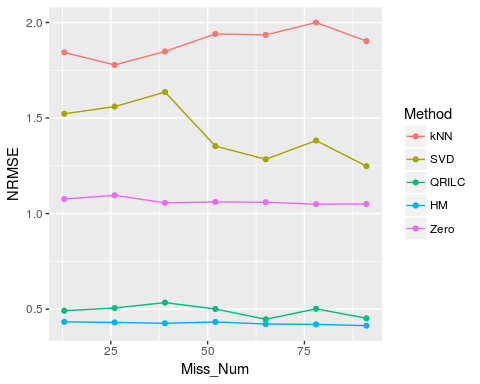


**Figure S5:** NRMSE for 5 different imputation methods on MNAR. X-axis is the number of missing variables and y-axis is the NRMSE.

**Table S10:** NRMSE for 5 different imputation methods on MNAR.

| kNN | SVD | QRILC | HM | Zero | Miss_Prop | Miss_Num |
| --- | --- | --- | --- | --- | --- | --- |
| 1.843693 | 1.522403 | 0.4919585 | 0.4334591 | 1.076480 | 0.0493007 | 13 |
| 1.777751 | 1.559842 | 0.5060922 | 0.4305481 | 1.095724 | 0.0870629 | 26 |
| 1.848609 | 1.636142 | 0.5341109 | 0.4258740 | 1.056053 | 0.1394328 | 39 |
| 1.940268 | 1.353173 | 0.5008561 | 0.4330214 | 1.061102 | 0.1832945 | 52 |
| 1.935096 | 1.284224 | 0.4469191 | 0.4221009 | 1.059489 | 0.2303030 | 65 |
| 2.000223 | 1.382448 | 0.5017534 | 0.4202394 | 1.049319 | 0.2719503 | 78 |

**Table S11:** The melted table for Table S10.

| Miss_Num | Method | NRMSE |
| --- | --- | --- |
| 13 | kNN | 1.843693 |
| 26 | kNN | 1.777751 |
| 39 | kNN | 1.848609 |
| 52 | kNN | 1.940268 |
| 65 | kNN | 1.935096 |
| 78 | kNN | 2.000223 |

The above melted table is for ggplot2

## MNAR NRMSE Rank evaluation and plot

MNAR_NRMSE_rank_list <- NRMSE_rank_cal_plot(MNAR_list, plot = T, x = 'Miss_Num')

## [1] 1
## [1] 2
## [1] 3
## [1] 4
## [1] 5
## [1] 6
## [1] 7


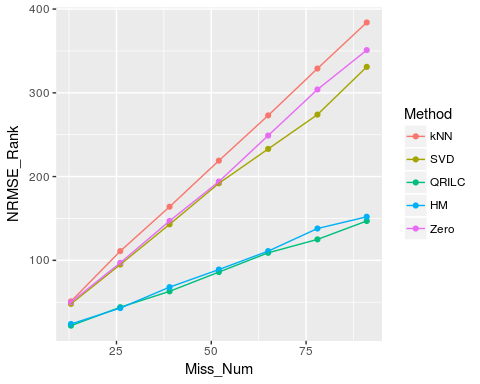


**Figure S6:** NRMSE-based Sum of Ranks (SOR) for 5 different imputation methods on MNAR. X-axis is the number of missing variables and y-axis is the SOR.

**Table S12:** NRMSE-based Sum of Ranks (SOR) for 5 different imputation methods on MNAR.

| kNN | SVD | QRILC | HM | Zero | Miss_Prop | Miss_Num |
| --- | --- | --- | --- | --- | --- | --- |
| 51 | 48 | 22 | 24 | 50 | 0.0493007 | 13 |
| 111 | 95 | 44 | 43 | 97 | 0.0870629 | 26 |
| 164 | 143 | 63 | 68 | 147 | 0.1394328 | 39 |
| 219 | 192 | 86 | 89 | 194 | 0.1832945 | 52 |
| 273 | 233 | 109 | 111 | 249 | 0.2303030 | 65 |
| 329 | 274 | 125 | 138 | 304 | 0.2719503 | 78 |

**Table S13:** The melted table of Table S12.

| Miss_Num | Method | NRMSE_Rank |
| --- | --- | --- |
| 13 | kNN | 51 |
| 26 | kNN | 111 |
| 39 | kNN | 164 |
| 52 | kNN | 219 |
| 65 | kNN | 273 |
| 78 | kNN | 329 |

The above melted table is for ggplot2

## NRMSE and SOR on MNAR

MNAR is truncated distributed in the dataset that might lead to unstable and biased parameter estimations using NRMSE directly, especially when missing variables are within different numerically ranges. Thus, we introduced a new evaluation criterion, NRMSE-based SOR, in this work. Here, we randomly (1) chose 40% variables as missing variables (n=52), (2) generated a random quantile cut off from the range 30%~60% uniformly for each of these missing variables, and (3) replaced those elements under the cutoff with missing values. Then we compared the NRMSE and NRMSE-based cumulative Ranks across these missing variables.


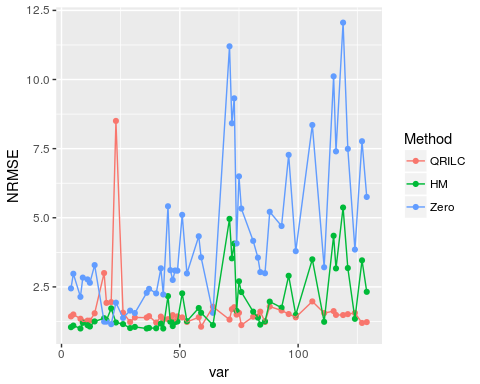


**Figure S7:** NRMSE of three MNAR imputation methods across each missing variable. X-axis is the missing variable index and y-axis is the NRMSE for this missing variable.

Zero shows overall unstable and poor performance while QRILC shows overall stable and good performances except for one outlier variable.


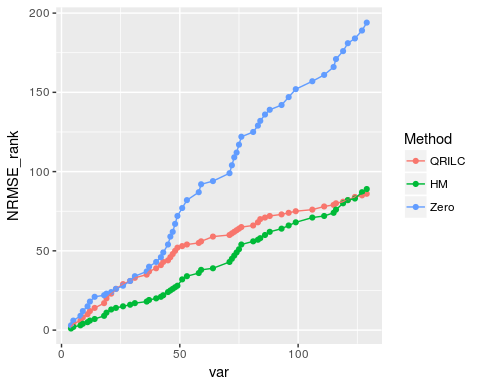


**Figure S8:** NRMSE-based cumulative Ranks of three MNAR imputation methods across each missing variable. X-axis is the missing variable index and y-axis is the NRMSE-based cumulative Ranks. Cumulative Ranks showed more robust performances compared to the NRMSE method.

## PCA Procrustes analysis on MNAR

MNAR_PCA_ProSS_list <- Procrustes_cal_plot(MNAR_list, DR = 'PCA', nPCs = 2, x = 'Miss_Num', plot = T)

## [1] 1
## [1] 2
## [1] 3
## [1] 4
## [1] 5
## [1] 6
## [1] 7


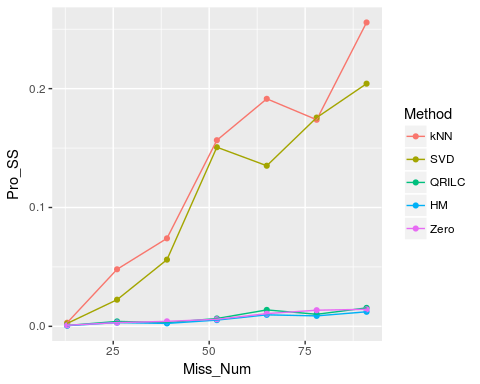


**Figure S9:** The PCA-Procrustes Sum of Squared Error of 5 different imputation methods on MNAR. X-axis is the number of missing variables and y-axis is the PCA-Procrustes Sum of Squared Error.

**Table S14:** The PCA-Procrustes Sum of Squared Error of 5 different imputation methods on MNAR.

| kNN | SVD | QRILC | HM | Zero | Miss_Prop | Miss_Num |
| --- | --- | --- | --- | --- | --- | --- |
| 0.0030569 | 0.0024599 | 0.0007304 | 0.0005824 | 0.0008678 | 0.0493007 | 13 |
| 0.0480066 | 0.0224202 | 0.0041478 | 0.0031385 | 0.0031507 | 0.0870629 | 26 |
| 0.0740282 | 0.0561170 | 0.0031186 | 0.0024640 | 0.0042093 | 0.1394328 | 39 |
| 0.1565980 | 0.1507121 | 0.0066117 | 0.0052507 | 0.0059363 | 0.1832945 | 52 |
| 0.1914342 | 0.1351302 | 0.0138310 | 0.0096825 | 0.0107177 | 0.2303030 | 65 |
| 0.1738227 | 0.1756455 | 0.0101566 | 0.0088044 | 0.0135821 | 0.2719503 | 78 |

**Table S15:** The melted table of Table S14

| Miss_Num | Method | Pro_SS |
| --- | --- | --- |
| 13 | kNN | 0.0030569 |
| 26 | kNN | 0.0480066 |
| 39 | kNN | 0.0740282 |
| 52 | kNN | 0.1565980 |
| 65 | kNN | 0.1914342 |
| 78 | kNN | 0.1738227 |

The above melted table is for ggplot2

## MNAR T-test results correlation

MNAR_Ttest_cor_list <- Ttest_cor_cal_plot(MNAR_list, group = group, plot = T, x = 'Miss_Num', cor = 'P')

## [1] 1
## [1] 2
## [1] 3
## [1] 4
## [1] 5
## [1] 6
## [1] 7


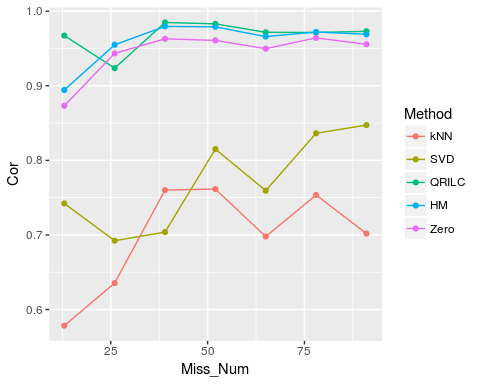


**Figure S10:** The Pearson Correlation of log-transformed P-values from student t-test between imputed data and complete data of missing variables on MNAR. X-axis is the number of missing variables and y-axis is the Pearson’s correlation.

**Table S16:** The Pearson Correlation of log T-test P-values between imputed data and complete data of missing variables on MNAR.

| kNN | SVD | QRILC | HM | Zero | Miss_Prop | Miss_Num |
| --- | --- | --- | --- | --- | --- | --- |
| 0.5784013 | 0.7421768 | 0.9672143 | 0.8943420 | 0.8732141 | 0.0493007 | 13 |
| 0.6354761 | 0.6922887 | 0.9235852 | 0.9549122 | 0.9433824 | 0.0870629 | 26 |
| 0.7601306 | 0.7038311 | 0.9848421 | 0.9795935 | 0.9629277 | 0.1394328 | 39 |
| 0.7615114 | 0.8150958 | 0.9829798 | 0.9789519 | 0.9607697 | 0.1832945 | 52 |
| 0.6978657 | 0.7593904 | 0.9716515 | 0.9658767 | 0.9497483 | 0.2303030 | 65 |
| 0.7535756 | 0.8363294 | 0.9713824 | 0.9720855 | 0.9641362 | 0.2719503 | 78 |

**Table S17:** The Spearman Correlation of log T-test P-values between imputed data and complete data of missing variables on MNAR.

| kNN | SVD | QRILC | HM | Zero | Miss_Prop | Miss_Num |
| --- | --- | --- | --- | --- | --- | --- |
| 0.3901099 | 0.2362637 | 0.9285714 | 0.9725275 | 0.9505495 | 0.0493007 | 13 |
| 0.6348718 | 0.5829060 | 0.9589744 | 0.9254701 | 0.9295726 | 0.0870629 | 26 |
| 0.7064777 | 0.6530364 | 0.9777328 | 0.9672065 | 0.9506073 | 0.1394328 | 39 |
| 0.5924187 | 0.6535473 | 0.9692649 | 0.9651669 | 0.9531290 | 0.1832945 | 52 |
| 0.5629808 | 0.6408217 | 0.9640297 | 0.9696241 | 0.9615385 | 0.2303030 | 65 |
| 0.6645380 | 0.7563702 | 0.9702070 | 0.9765551 | 0.9713957 | 0.2719503 | 78 |

## MNAR PLS Procrustes analysis and plot

MNAR_PLS_ProSS_list <- Procrustes_cal_plot(MNAR_list, DR = 'PLS', nPCs = 2, outcome = group, x = 'Miss_Num', plot = T)

## [1] 1
## [1] 2
## [1] 3
## [1] 4
## [1] 5
## [1] 6
## [1] 7


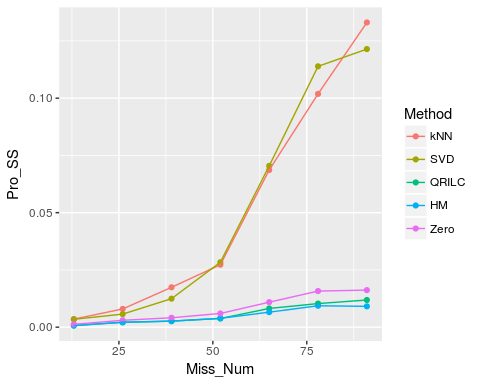


**Figure S11:** The PLS-DA-Procrustes Sum of Squared Error of 5 different imputation methods on MNAR. X-axis is the number of missing variables and y-axis is the PLS-Procrustes Sum of Squared Error.

**Table S18:** The PLS-DA-Procrustes Sum of Squared Error of 5 different imputation methods on MNAR.

| kNN | SVD | QRILC | HM | Zero | Miss_Prop | Miss_Num |
| --- | --- | --- | --- | --- | --- | --- |
| 0.0035220 | 0.0034429 | 0.0006686 | 0.0008045 | 0.0012759 | 0.0493007 | 13 |
| 0.0079679 | 0.0057215 | 0.0021548 | 0.0021177 | 0.0030167 | 0.0870629 | 26 |
| 0.0174182 | 0.0124557 | 0.0026718 | 0.0026206 | 0.0041173 | 0.1394328 | 39 |
| 0.0272772 | 0.0283593 | 0.0037994 | 0.0038734 | 0.0060006 | 0.1832945 | 52 |
| 0.0686296 | 0.0704292 | 0.0081831 | 0.0065649 | 0.0109262 | 0.2303030 | 65 |
| 0.1018619 | 0.1138955 | 0.0103098 | 0.0093510 | 0.0157715 | 0.2719503 | 78 |

**Table S19:** The melted table of Table S18.

| Miss_Num | Method | Pro_SS |
| --- | --- | --- |
| 13 | kNN | 0.0035220 |
| 26 | kNN | 0.0079679 |
| 39 | kNN | 0.0174182 |
| 52 | kNN | 0.0272772 |
| 65 | kNN | 0.0686296 |
| 78 | kNN | 0.1018619 |

The above melted table is for ggplot2


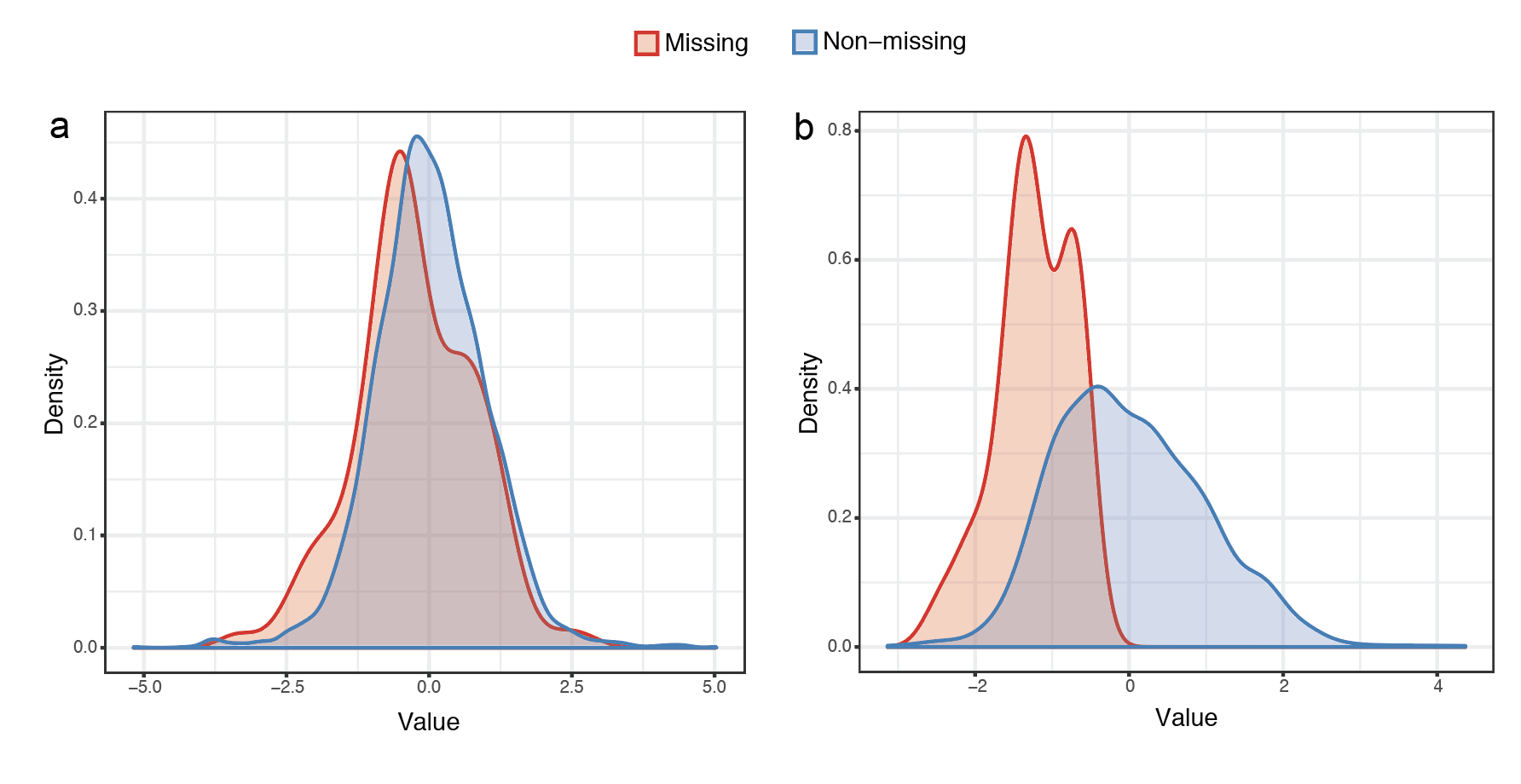
**Figure S12a:** The density plots of the retrieved missing part and the non-missing part on GC-MS untargeted dataset; **Figure S12b:** The density plots of elements that have response value but fail to be quantified and the non-missing response part on LC-MS targeted dataset. X-axis is the value (scaled) of elements and y-axis is the kernel density.

**Table S20:** The NRMSE and PCA-Procrustes Sum of Squared Error of 5 different imputation methods on GC-MS untargeted dataset.

|  | kNN | SVD | Mean | Median | RF |
| --- | --- | --- | --- | --- | --- |
| NRMSE | 1.178464 | 1.168489 | 1.293404 | 1.263816 | 1.153475 |
| PCA-Procrustes Sum of Squared Error | 0.004964 | 0.005258 | 0.005593 | 0.00547 | 0.004413 |

**Table S21:** The SOR and PCA-Procrustes Sum of Squared Error of 5 different imputation methods on LC-MS targeted dataset.

|  | kNN | SVD | QRILC | HM | Zero |
| --- | --- | --- | --- | --- | --- |
| SOR | 28 | 26 | 10 | 9 | 17 |
| PCA-Procrustes Sum of Squared Error | 0.01875 | 0.01191 | 0.0001843 | 0.0003221 | 0.003164 |

**Note**: For all tables in this vignette, only the head (first parts of the data) was printed.
